# Utilization of a UPLC-MS/MS Approach to Elucidate the Role of ABCB1-Mediated Paclitaxel Resistance in Non-Small Cell Lung Cancer Cells

**DOI:** 10.32604/or.2025.068967

**Published:** 2026-01-19

**Authors:** Sha Hu, Wenjing Wang, Qianfang Hu, Rujuan Zheng, Qinghe Huang, Hui Shi, Xinyuan Ding, Wenjuan Wang, Zengyan Zhu

**Affiliations:** 1 Department of Pharmacy, Children’s Hospital of Soochow University, Suzhou, 215025, China; 2 College of Pharmaceutical Sciences, Soochow University, Suzhou, 215123, China; 3 Department of Pharmacy, The Affiliated Suzhou Hospital of Nanjing Medical University, Suzhou, 215001, China

**Keywords:** Non-small cell lung cancer (NSCLC), chemoresistance, paclitaxel, ATP-binding cassette subfamily B member 1 (ABCB1), ultra performance liquid chromatography-tandem mass spectrometry (UPLC-MS/MS)

## Abstract

**Objectives:**

Acquired resistance to paclitaxel represents a critical barrier to the effective chemotherapy of non-small cell lung cancer (NSCLC). The present study aimed to elucidate the molecular and pharmacological mechanisms promoting paclitaxel resistance in NSCLC and to explore potential strategies for overcoming this resistance.

**Methods:**

Here, we report an integrated pharmacological and analytical approach to quantify paclitaxel disposition and overcome resistance in a A549/TAX cell model (paclitaxel-resistant A549 cells).

**Results:**

Cell counting kit-8 (CCK-8) assay, colony formation, and apoptosis assays confirmed that A549/TAX cells exhibited marked resistance to paclitaxel relative to parental A549 cells. Based on transcriptome profiling by RNA sequencing analysis and validation by western blotting assay, we found that the expression of the ATP-binding cassette subfamily B member 1 (ABCB1) (the encoded protein is termed P-glycoprotein) was significantly upregulated in resistant cells. By using ultra performance liquid chromatography-tandem mass spectrometry (UPLC-MS/MS), we demonstrated that ABCB1 overexpression promotes enhanced efflux of intracellular paclitaxel, thereby lowering its cytotoxic accumulation. Genetic silencing of ABCB1 or pharmacological inhibition with the specific P-glycoprotein modulator elacridar or tariquidar restored intracellular paclitaxel levels, as determined by UPLC-MS/MS, and synergistically decreased cell viability as observed in CCK-8 assay.

**Conclusion:**

These findings reveal that the ABCB1-mediated drug efflux is a crucial mechanism underlying paclitaxel resistance in NSCLC cells, with UPLC-MS/MS serving as a sensitive analytical method to detect paclitaxel concentration. Inhibition of ABCB1 is a promising therapeutic strategy to resensitize resistant tumor cells to paclitaxel.

## 1 Introduction

Lung cancer is one of the most prevalent and lethal malignancies worldwide [1]. Non-small cell lung cancer (NSCLC), accounting for 80–85% of all lung cancer cases, is characterized by high invasiveness, a high recurrence rate, and poor therapeutic outcomes [2]. Although several treatment options are available for NSCLC, including surgical intervention, radiotherapy, chemotherapy, molecular targeted therapy, and immunotherapy [3,4], chemotherapy remains the principal therapeutic approach for NSCLC because of its broad-spectrum efficacy. Among various chemotherapeutic agents, paclitaxel is widely recognized as the first-line treatment for NSCLC [5]. Notably, paclitaxel when combined with immunotherapy shows remarkable efficacy for treating advanced lung cancers, particularly NSCLC lacking driver mutation genes, lung adenocarcinoma, and lung squamous cell cancer [6,7].

Paclitaxel is originally derived from the bark of *Taxus brevifolia* [8,9]. It functions as a microtubule stabilizer by specifically binding to tubulin proteins. This interaction stabilizes the microtubules, thereby reducing their dynamic instability and inhibiting the normal processes of depolymerization and polymerization [10]. Despite its initial effectiveness, long-term administration of paclitaxel often induces the development of acquired resistance, ultimately diminishing its therapeutic efficacy [11]. Consequently, there is a critical need to elucidate the molecular regulatory network underlying the emergence of paclitaxel resistance and identify novel targets for its reversal.

Numerous factors have been attributed to the development of paclitaxel resistance: (1) the expression and activity of ATP-binding cassette family members involved in multidrug efflux transporters; (2) an imbalance between apoptotic proteins and antiapoptotic proteins [12]; (3) the maintenance of stemness mediated by cancer stem cells [13]; and (4) alterations in microtubules and tubulins [14,15]. Among these factors, the emergence of multidrug resistance mediated by ATP-binding cassette subfamily B member 1 (ABCB1) represents the most crucial barrier to chemotherapy efficacy and has been reported in various cancers, including colorectal cancer and breast cancer [16,17].

A variety of ABCB1 inhibitors have been developed to address chemotherapeutic drug resistance induced by ABCB1 overexpression in cancer cells [18–20]. Inhibitors such as verapamil, tariquidar, and elacridar have been investigated as potential agents to overcome multidrug resistance in cancer therapy by inhibiting the efflux of chemotherapeutic drugs mediated by the ABCB1 transporter [21–23]. However, their clinical application is limited because of poor specificity, off-target effects, and substantial toxicity [24]. Currently, there is also a lack of data related to paclitaxel concentration detection to substantiate the role of ABCB1 in drug efflux. Additionally, the efficacy of ABCB1 inhibitors in reversing chemotherapy resistance remains inconclusive, as there is insufficient robust clinical evidence to support their ability to improve therapeutic outcomes.

Given the vital role of ABCB1 in mediating paclitaxel resistance, it is important to directly quantify paclitaxel levels in NSCLC cells to better understand the underlying mechanisms. Intracellular and extracellular drug concentrations can serve as indicators of resistance and provide insights into ABCB1-mediated drug efflux. To address this issue, we designed a study in which ultra-performance liquid chromatography-tandem mass spectrometry (UPLC-MS/MS) was used to precisely measure paclitaxel concentration. This approach enables to investigate how ABCB1 expression affects cellular sensitivity to paclitaxel and evaluate the potential of ABCB1 inhibitors in overcoming drug resistance. Overall, the present study aimed to establish a robust analytical and pharmacological framework for assessing paclitaxel disposition and guiding the development of targeted strategies to resensitize resistant NSCLC cells.

## 2 Materials and Methods

### 2.1 Cell Culture

Human lung adenocarcinoma A549 cells were cultured in Dulbecco’s Modified Eagle’s Medium supplemented with 10% fetal bovine serum and 1% penicillin-streptomycin (Gibco, Grand Island, NY, USA). A549/TAX cells (paclitaxel-resistant A549 cells), purchased from Fuheng Biology Co., Ltd. (Shanghai, China), were maintained in Ham’s F-12K medium (Sangon, Shanghai, China) containing 10% FBS (Gibco Life Technologies, Waltham, MA, USA) and 200 ng/mL paclitaxel (MedChemExpress, Shanghai, China; Cat. No. HY-B0015). Both A549 and A549/TAX cells were incubated at 37°C with 5% CO_2_. All cell lines were authenticated by short tandem repeat profiling (conducted by Fuheng Biology Co., Ltd.) and were confirmed to be free of mycoplasma contamination by using a PCR-based detection kit (Applied Biological Materials, Richmond, BC, Canada; Cat. No. G238).

### 2.2 Cell Counting Kit-8 (CCK-8) Assay

A total of 2 × 10^3^ A549 and A549/TAX cells per well were seeded onto 96-well plates. After 24 h, serial concentrations (0.01~16,200 ng/mL) of paclitaxel were added to the wells. Following a 48-h incubation period, 10% CCK-8 reagent (Dojindo, Tokyo, Japan; Cat. No. CK04) was added to each well, and the plates were incubated at 37°C in the dark for 2–3 h. Absorbance was measured at 450 nm using a microplate reader (BioTek Synergy H1, Winooski, VT, USA). The half-maximal inhibitory concentration (IC_50_) values were determined by fitting dose-response curves using GraphPad Prism (v10.2.1) (GraphPad Software, San Diego, CA, USA) software.

### 2.3 Colony Formation Assay

A total of 8 × 10^2^ A549 and A549/TAX cells per well were plated onto 6-well plates and cultured in complete medium, and the culture medium was replaced every 3 days. On the third day of cell culture, paclitaxel (1.6/8/40 μg/mL) or ABCB1 inhibitors (25 ng/mL tariquidar (MedChemExpress, Shanghai, China; Cat. No. HY-10550) or 1.5 μg/mL elacridar (MedChemExpress, Shanghai, China; Cat. No. HY-50879)) were added. After 14 days, colonies were fixed with 4% paraformaldehyde (Servicebio, Wuhan, China) for 40 min, stained with 0.1% crystal violet solution (Biosharp, Hefei, China) for 40 min, and washed with water. Colony numbers were quantified using ImageJ software, version 1.54 (National Institutes of Health, Bethesda, MD, USA).

### 2.4 Apoptosis Assay

A total of 5 × 10^5^ A549 and A549/TAX cells per well were seeded onto 6-well plates with complete medium. After 24 h, paclitaxel (1 μg/mL) alone or combined with ABCB1 inhibitors (25 ng/mL tariquidar or 1.5 μg/mL elacridar) was added, and the cells were incubated for additional 48 h. Both floating and adherent cells were collected and stained with Annexin V-FITC and propidium iodide (Elabscience, Wuhan, China; E-CK-A211), and apoptosis levels were determined by flow cytometry using a BD FACSDiva software v9.1 (BD Biosciences, San Jose, CA, USA). The apoptosis rate was calculated with FlowJo v10.8.1 software.

### 2.5 RNA Sequencing (RNA-Seq) Analysis

A549 and A549/TAX cells (7 × 10^6^ cells per condition) were harvested in triplicate. Total RNA was extracted using TRIzol reagent (Invitrogen, Carlsbad, CA, USA; Cat. No. 15596026) and sent to Azenta Life Sciences for further sequencing and analysis.

### 2.6 Western Blotting (WB) Assay

A549 and A549/TAX cells were lysed with RIPA buffer (Beyotime, Shanghai, China) containing a protease inhibitor cocktail (Roche, Basel, Switzerland). Protein concentrations were determined using the bicinchoninic acid assay (Invitrogen, CA, USA; Cat. No. A55864). The total protein was separated by sodium dodecyl sulfate-polyacrylamide gel electrophoresis and transferred to polyvinylidene fluoride (Millipore, Darmstadt, Germany) membrane activated with methanol. The membrane was blocked with 5% bovine serum albumin at room temperature and incubated with primary antibodies (ABCB1, 1:1000, Abcam, Cambridge, UK; Cat. No. ab170904. GAPDH (1:2000, Cat. No. 60004-1-Ig), ABCG2 (1:1000, Cat. No. 27286-1-AP), SOX2 (1:1000, Cat. No. 20118-1-AP), Proteintech, Wuhan Sanying Biotechnology Co., Ltd., Wuhan, China. ABCC1, 1:1000, ABchlnal, Wuhan, China; Cat. No. A2223. β-catenin, 1:1000, Santa Cruz, CA, USA; Cat. No. sc-7963) overnight at 4°C. On the second day, the membrane was washed with Tris-buffered saline containing Tween-20 and incubated with rabbit (1:3000, Cell Signaling Technology, Danvers, MA, USA; Cat. No. 7074) or mouse (1:3000, Cell Signaling Technology, Danvers, MA, USA; Cat. No. 7076) secondary antibodies according to the manufacturer’s specifications. Protein bands were visualized using an enhanced chemiluminescence reagent (Invitrogen, CA, USA; Cat. No. 32106) and quantified with ImageJ software (v1.54).

### 2.7 Transient Knockdown Assay

A549/TAX cells were seeded into 6-well plates with the appropriate number of 3 × 10^5^/well. After the cells reached 60%~80% confluence, a transfection mixture containing 300 μL Opti-MEM (Gibco, Grand Island, NY, USA), 9 μL Lipofectamine RNAiMAX (Thermo, Waltham, MA, USA), and 30 pmol siRNA was added to each well in accordance with the manufacturer’s protocol. The siRNA sequences of ABCB1 were as follows:

siABCB1-negtive control: 5^′^-UUCUCCGAACGUGUCACGU-3^′^;

siABCB1-1: 5^′^-CGACAGAAUAGUAACUUGUUU-3^′^;

siABCB1-2: 5^′^-GCAGCAAUUAGAACUGUGAUU-3^′^;

siABCB1-3: 5^′^-CCGAACACAUUGGAAGGAAAU-3^′^.

After 3 days, the cells were collected to prepare for further functional assays.

### 2.8 Quantitative Reverse Transcription PCR (qRT-PCR)

Total RNA from A549 and A549/TAX cells was obtained using the RNA-Quick Purification kit (YiShan Biotech, Shanghai, China; Cat. No. RN001). The reverse transcription-PCR reaction system comprised 4 μL of 5× PrimeScript Buffer, 1 μL of PrimeScript RT Enzyme Mix, 1 μL of Oligo dT Primer, 1 μL of Random 6mers, and 1 μg of RNA; the final reaction volume was adjusted to 20 μL. Quantitative real-time PCR was conducted using the PrimeScript RT Reagent Kit (Takara, Shimogyo-ku, Kyoto, Japan; Cat. No. RR037A). The PCR primers of ABCB1 and GAPDH were as follows:

ABCB1 forward: 5^′^-GGGAGCTTAACACCCGACTTA-3^′^;

ABCB1 reverse: 5^′^-GCCAAAATCACAAGGGTTAGCTT-3^′^;

GAPDH forward: 5^′^-GGAGCGAGATCCCTCCAAAAT-3^′^;

GAPDH reverse: 5^′^-GGCTGTTGTCATACTTCTCATGG-3^′^.

The relative gene expression was calculated for each sample based on cycle threshold values.

### 2.9 Ultra Performance Liquid Chromatography-Tandem Mass Spectrometry (UPLC-MS/MS)

#### 2.9.1 Reagents and Chemicals

Paclitaxel and paclitaxel-D5 were purchased from First Standard® of Alta Scientific Co., Ltd. (Tianjin, China; Cat. No. 1ST000431D5). Paclitaxel-D5 was used as an isotope-labeled internal standard (IS) to ensure accurate and reliable quantification of paclitaxel. High Performance Liquid Chromatography (HPLC)-grade methanol (Cat. No. 34860) and acetonitrile (Cat. No. 34851) were purchased from Sigma-Aldrich (St. Louis, MO, USA). Deionized water was obtained by filtration through a Milli-Q system (Millipore, Milford, MA, USA).

#### 2.9.2 Preparation of Stock Solutions, Calibration Standards, and Quality Control Samples

Standard stock solutions of paclitaxel and isotope-labeled IS stock solutions of paclitaxel-D5 were dissolved in methanol to yield concentrations of 1.0 mg/mL for paclitaxel and paclitaxel-D5, and the solutions were stored at −80°C. A series of paclitaxel standard solutions with concentrations of 10.0, 50.0, 100.0, 500.0, 1000.0, 5000.0, 10,000.0, and 50,000.0 ng/mL and a quality control (QC) standard solution with concentrations of 10.0, 1000.0, and 40,000.0 ng/mL were prepared by quantitative dilution of a water-methanol solution (50:50) and stored at −80°C for later use. Paclitaxel-D5 stock solutions were quantitatively diluted with a water-methanol solution (50:50) to prepare an IS solution with a mass concentration of 1000.0 ng/mL (hereinafter referred to as IS), which was stored at −80°C for later use.

#### 2.9.3 Sample Preparation

Liquid-liquid extraction was used to extract paclitaxel from the samples. Briefly, 100 μL of the sample was spiked with 10 μL IS, and 1 mL of methyl tert-butyl ether was then added. This mixture was vortexed for 5 min. Following centrifugation (Thermo Fisher Scientific, Model 5424 R, Waltham, MA, USA) at 14,000 rpm for 10 min, 900 μL of the upper organic layer was transferred into a tube and evaporated to dryness under a nitrogen stream. The residue was reconstituted in 100 μL mobile phase (water: methanol, 20:80, v/v), and 5 μL of the supernatant was injected into the UPLC-MS/MS system.

#### 2.9.4 Instrument and UPLC-MS/MS Conditions

UPLC-MS/MS analysis was performed on the Xevo TQD system (Waters Corp., Milford, MA, USA), equipped with the ACQUITY UPLC I-Class system.

A. Chromatographic conditions

Column: ACQUITY UPLC BEH C18 column (2.1 mm × 50 mm, 1.7 μm; Waters Corp.). Mobile phase: phase A: H_2_O (0.1% formic acid), phase B: methanol. Gradient elution procedure: 0–0.2 min, 90% A, 0.2–1 min, 90% A→20% A; 1–2 min, 20% A; 2.0–2.1 min, 20% A→90% A; 2.1–3.0 min, 90% A. Flow rate: 0.3 mL/min. Column temperature: 40°C. Injection volume: 5 μL.

B. Mass spectrometry conditions

Electrospray ionization source, multiple reaction monitoring mode scanning, and positive ion mode detection. Paclitaxel: m/z 854.2.0 → 286.0, cone voltage = 30 V, collision voltage = 15 V, and scanning time = 0.163 s. Paclitaxel-D5: m/z 859.2 → 291.2, cone voltage = 30 V, collision voltage = 20 V, and scanning time = 0.163 s.

#### 2.9.5 UPLC-MS/MS Method Validation

The methods for quantifying paclitaxel concentration in the cell lysate and medium mixture were validated according to the current US Food and Drug Administration guidelines for bioanalytical method validation (Docket Number: FDA-2013-D-1020). The validation experiments included determination of specificity, lower limit of quantification (LLOQ), accuracy, precision, carry-over, and stability and generation of a calibration curve. According to the regulations, three lots of spiked samples at low, middle, and high QCs in five replicates were selected and tested. The accuracy and precision of the determination method were estimated based on relative error (RE) and relative standard deviation (RSD), respectively.

### 2.10 Preparation and Processing of Cells

A549 and A549/TAX cells were seeded into 6-well plates with the appropriate number of 5 × 10^5^/well. After 24 h, the cells were treated with ABCB1 inhibitors (25 ng/mL tariquidar or 1.5 μg/mL elacridar) combined with various concentrations (1/2/4 μg/mL) of paclitaxel and subsequently co-incubated for 2 days. The culture medium was then collected and designated as the cell supernatant. Following 0.25% trypsinization, the adherent cells were harvested and resuspended in PBS (1×, pH: 7.4, Sangon, Shanghai, China; Cat. No. E607008) to a volume equivalent to that of the cell supernatant. Finally, the cell pellets were subjected to three cycles of rapid freezing in liquid nitrogen, followed by thawing at room temperature; the cell pellets were ultrasonicated (Scientz-IID, Scientz Biotechnology Co., Ltd., Ningbo, China) in an ice bath (10 s pulses with a 5 s interval) and subsequently stored at 4°C.

### 2.11 Clinical Samples

NSCLC tumor tissues (1.0 cm^3^) were obtained after written informed consent from patients at the Affiliated Suzhou Hospital of Nanjing Medical University. All procedures were approved by the Ethics Committee of the Affiliated Suzhou Hospital of Nanjing Medical University (Approval No. K-2024-035-K01) and conducted in accordance with the Declaration of Helsinki.

### 2.12 Immunohistochemical (IHC) Assay

The tumor samples were fixed with 10% neutral-buffered formalin, paraffin-embedded, and sectioned at 4 μm thickness. The sections were then deparaffinized, rehydrated, and subjected to antigen retrieval in citrate buffer (pH 6.0). Endogenous peroxidase activity was blocked with 3% H_2_O_2_ for 10 min, followed by blocking of nonspecific binding. The slides were initially incubated overnight at 4°C with primary antibodies (ABCB1, 1:200, Abcam, Cambridge, UK; Cat. No. ab170904), subsequently incubated with HRP-conjugated secondary antibodies (goat anti-rabbit IgG-HRP, 1:50, Beyotime Biotechnology, Shanghai, China; Cat. No. A0208), and visualized using 3,3^′^-diaminobenzidine (DAB) substrate solution (0.05% DAB and 0.03% H_2_O_2_ in PBS; Beyotime, Shanghai, China). Finally, the sections were counterstained with 0.1% hematoxylin for 1 min, rinsed with running tap water, dehydrated through graded ethanol solutions (70%, 85%, 95%, and 100%), cleared in xylene, and mounted with neutral resin.

### 2.13 Statistical Analysis

All numerical data are expressed as mean ± standard deviation (SD). Statistical significance was evaluated using a *t*-test, one-way ANOVA, or two-way ANOVA. All statistical analyses were conducted using GraphPad Prism (v10.2.1). Based on the RNA-seq data, we identified differentially expressed genes (DEGs) and visualized them with a volcano plot. A heatmap of the DEGs was generated using the ‘pheatmap’ package (v1.0.12) to show their expression patterns. Pathway enrichment analysis was then performed on these DEGs with the ‘ClusterProfiler’ package (v4.6.0); significantly enriched pathways were presented as bubble charts using ‘ggplot2 (v3.4.2)’. All analyses were conducted in R (v4.3.3). *p* < 0.05 was considered statistically significant. Significance levels were indicated as follows: *p < 0.05, **p < 0.01, and ***p < 0.001.

## 3 Results

### 3.1 ABCB1 Is Identified as a Key Factor Promoting Paclitaxel Resistance in NSCLC Cells

**Figure 1:**
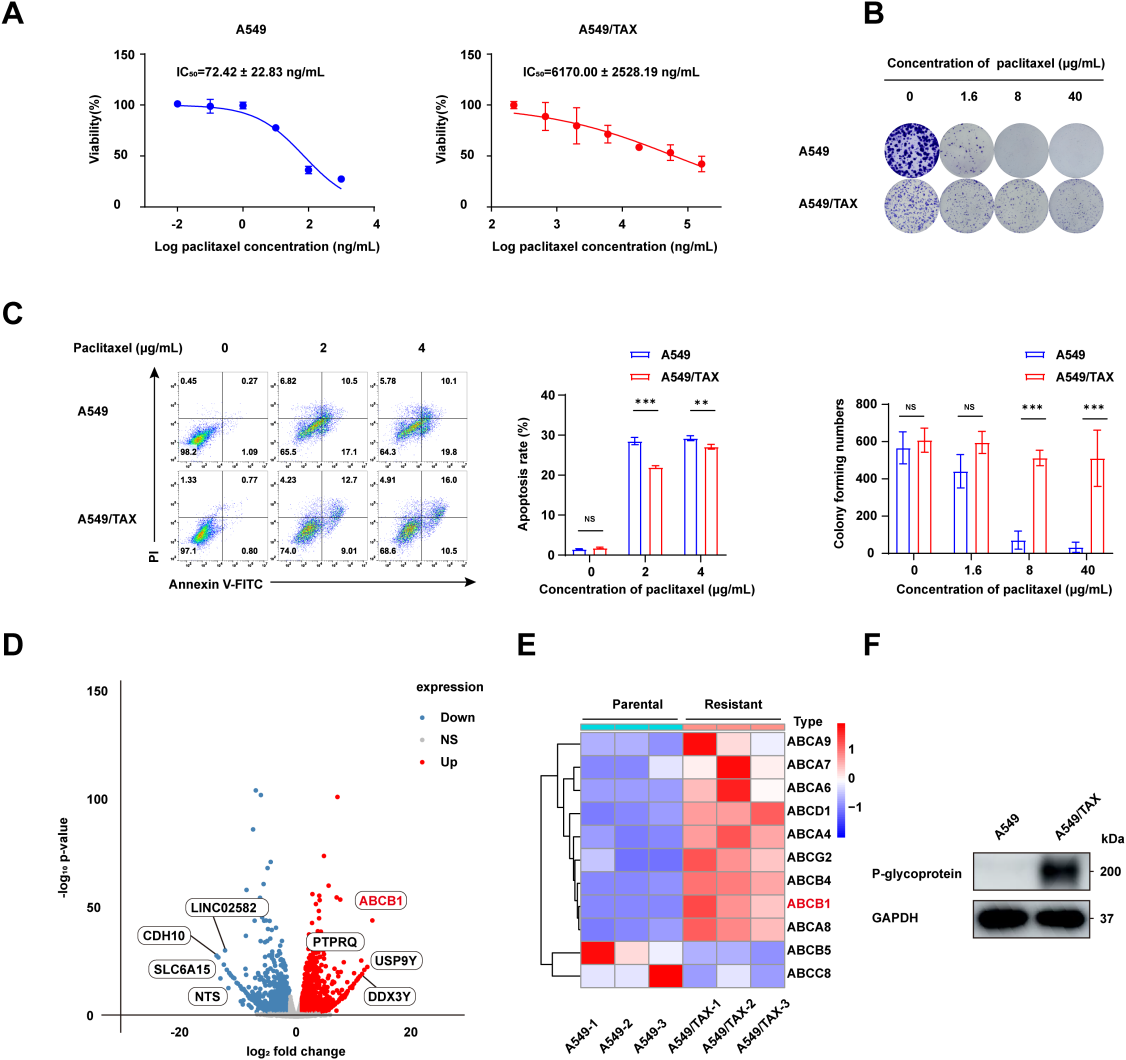
ABCB1 is identified as a key factor promoting paclitaxel resistance in NSCLC cells. (**A**) CCK-8 assay showing efficacy of paclitaxel in NSCLC cells (*n* = 3). (**B**) The difference in colony formation between A549 and A549/TAX cells (*n* = 3). (**C**) The apoptotic rate of A549 and A549/TAX cells at the same paclitaxel concentration as determined by flow cytometry (*n* = 3). (**D**) Volcano plot of differentially expressed genes. (**E**) Heatmap of the ABC family members in A549 and A549/TAX cells. (**F**) WB assay of P-glycoprotein expression in NSCLC cells (*n* = 3). **p < 0.01, ***p < 0.001. NS = not significant

**Figure A1:**
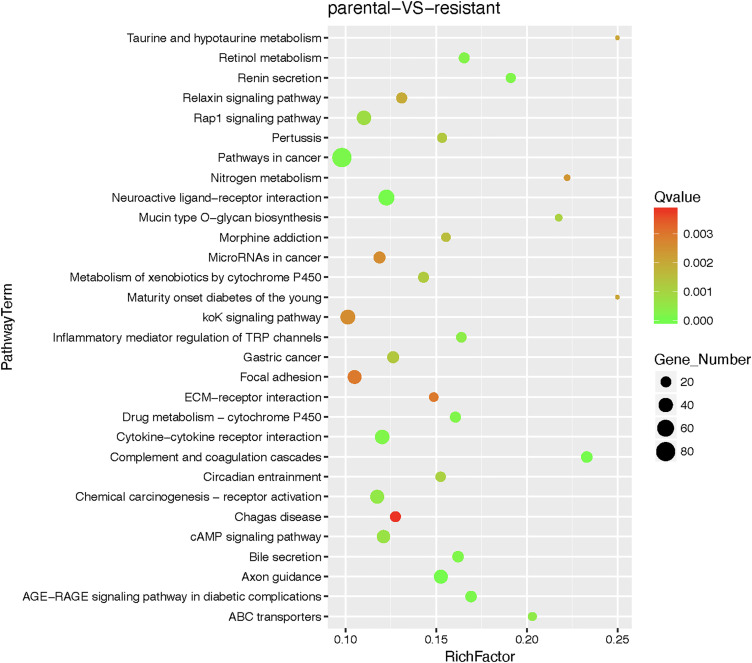
Bubble chart of pathway enrichment analysis results for parental cells (A549) and resistant cells (A549/TAX)

**Figure A2:**
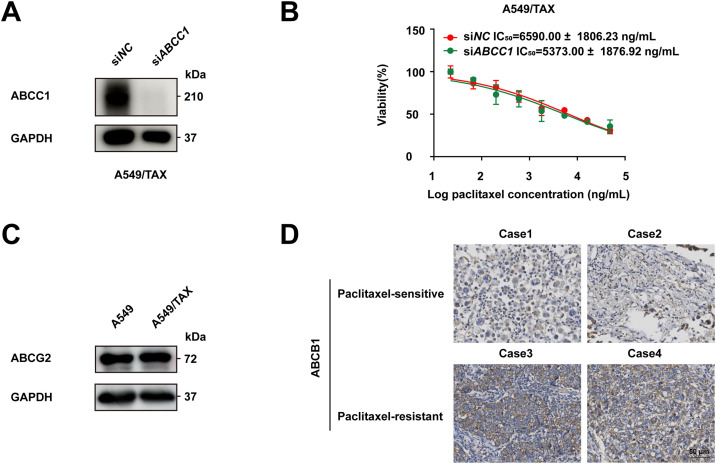
Roles of ABCB1, ABCC1 and ABCG2 in paclitaxel resistance in NSCLC. (**A**) The knockdown efficiency of ABCC1 in A549/TAX cells after knockdown by siRNA was verified by Western blot. (**B**) Cell viability of A549/TAX cells transfected with siNC or siABCC1 under treatment with various concentrations of paclitaxel was verified by the CCK-8 assay. (**C**) Western blot analysis comparing ABCG2 protein expression levels between A549 and A549/TAX cells. (**D**) Immunohistochemical staining of ABCB1 in clinical tissue samples of NSCLC. Case 1 and Case 2 were paclitaxel-sensitive NSCLC tissue. Case 3 and Case 4 were paclitaxel-resistant NSCLC tissues. Scale bar: 50 μm

To address the clinical challenge posed by paclitaxel resistance, we conducted experiments on a NSCLC cell line (A549) and a paclitaxel-resistant A549 cell line (A549/TAX). The paclitaxel sensitivity of both cell lines was assessed by a CCK-8 cell viability assay. The IC_50_ values for A549 and A549/TAX cells were 72.42 ± 22.83 and 6170.00 ± 2528.19 ng/mL, respectively (Fig. 1A). The resistance index was approximately 85, suggesting that A549/TAX cells possess moderate resistance to paclitaxel. The reliability of the A549/TAX cell line were further validated by colony formation and apoptosis assays. The colony formation assay revealed that at 0 μg/mL paclitaxel, A549 cells formed colonies with a larger individual area than that formed by A549/TAX cells, although the colony numbers of both cell lines were similar. At 1.6 μg/mL paclitaxel, the colony numbers of both cell lines did not differ significantly. However, at higher concentrations of paclitaxel (8 and 40 μg/mL), only A549 cells exhibited significantly reduced colony numbers compared to the A549/TAX group (Fig. 1B). Subsequently, we conducted apoptosis assays to examine the effect of paclitaxel on apoptosis levels in A549 and A549/TAX cells. In the absence of paclitaxel treatment, A549 and A549/TAX cells showed no significant difference in apoptosis levels. However, with an increase in the concentration of paclitaxel, a higher percentage of apoptotic cells was observed in A549 cells than in A549/TAX cells (Fig. 1C). To investigate the molecular mechanisms underlying the paclitaxel-resistant phenotype, we performed RNA-seq analysis on A549 and A549/TAX cells to identify genes associated with paclitaxel resistance. By applying a screening cutoff threshold (|log_2_FC| > 1 and *p* < 0.05), we identified 1268 DEGs showing a positive correlation with paclitaxel resistance. Notably, ABCB1 ranked first among these genes, with the highest log_2_FC value (Fig. 1D). The pathway enrichment analysis, presented as a bubble chart, revealed that the complement and coagulation cascades as well as ABC transporter pathways were most likely involved in paclitaxel resistance development (Fig. A1). We further examined the expression of other ABC transporter family members that contributed to this process (Fig. 1E). Previous studies have indicated that, in addition to ABCB1, both ABCC1 and ABCG2 are strongly associated with paclitaxel resistance in tumor cells [25,26]. To further clarify their roles, we detected the knockdown efficiency of ABCC1 by WB (Fig. A2A), then we conducted CCK-8 assays following ABCC1 knockdown combined with paclitaxel treatment; however, the results indicated that ABCC1 knockdown did not significantly increase sensitivity to paclitaxel (Fig. A2B). Additionally, WB assay of ABCG2 in A549 and A549/TAX cells demonstrated no significant differences in protein expression levels between the two cell lines (Fig. A2C). To confirm the results of RNA-seq analysis, we conducted WB assay and found that ABCB1 was markedly overexpressed in the A549/TAX cells compared to that in A549 cells (Fig. 1F). We also evaluated ABCB1 expression in specimens from NSCLC patients following paclitaxel treatment. IHC staining revealed that ABCB1 was highly expressed in paclitaxel-resistant NSCLC tissues (Fig. A2D). Thus, our data suggest that ABCB1 is a critical molecule involved in paclitaxel resistance shown by NSCLC cells, indicating potential for targeting this pathway in overcoming paclitaxel resistance.

### 3.2 UPLC-MS/MS Method Development and Validation

#### 3.2.1 Specificity

**Figure 2:**
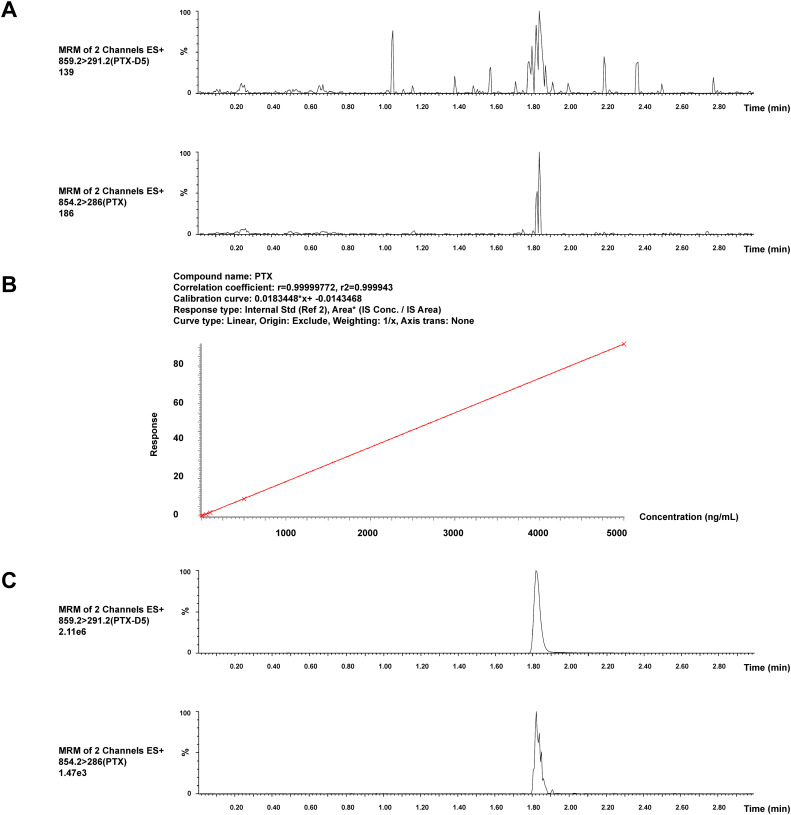
Chromatograms and calibration curve of paclitaxel and paclitaxel-D5. (**A**) Representative chromatograms of the pooled blank cellular matrix. (**B**) Calibration curve of paclitaxel, R^2^ > 0.999. (**C**) Representative chromatograms of the pooled blank cellular matrix spiked with an LLOQ sample

The retention times of paclitaxel and paclitaxel-D5 were 1.82 and 1.83 min, respectively. The interfering components in the pooled blank cellular matrix did not significantly influence analyte response, which was in accordance with the regulations. The blank is shown in Fig. 2A.

#### 3.2.2 Linearity and LLOQ

The calibration curve was plotted with the concentration ratio of paclitaxel and paclitaxel-D5 vs the nominal concentrations. The regression equation was fitted with the weighted least squares model (weight factor 1/X^2^), which achieved the best linear and least squares residuals. The linear range of paclitaxel was 15,000 ng/mL, and R^2^ (correlation coefficient) was greater than 0.999, which confirmed that the method has good linearity (Fig. 2B). The LLOQ of paclitaxel was 1 ng/mL (Fig. 2C).

#### 3.2.3 Precision and Accuracy

As shown in Table 1, both accuracy and precision of the determination method met the requirements of biological sample analysis.

**Table 1:** Intra-day and inter-day precision and accuracy of determining paclitaxel concentration (*n* = 5)

Analyte concentration (ng/mL)	Intra-day			Inter-day		
	Measured concentration (Mean ± SD)	Accuracy (RE%)	Precision (RSD%)	Measured concentration (Mean ± SD)	Accuracy (RE%)	Precision (RSD%)
1	1.07 ± 0.13	7.40	12.02	1.07 ± 0.12	6.60	11.07
100	99.40 ± 6.32	0.60	6.36	103.70 ± 4.85	3.70	4.67
4000	4039.90 ± 100.50	1.00	2.49	4033.50 ± 171.23	0.84	4.25

Note: SD, standard deviation; RE, relative error; RSD, relative standard deviation.

#### 3.2.4 Carry-Over

To assess the carry-over of the determination method, three double blank samples were injected directly after an upper limit of quantification sample. The first double blank response showed the absence of peaks (2.95% of the LLOQ sample) at the retention time of paclitaxel in plasma.

#### 3.2.5 Stability

The analyte showed good stability at low and high QC levels under the following conditions: 4 h at ambient temperature, three freeze-thaw cycles, and storage at −80°C for 30 days. No evident deviations were detected in the measured concentration of paclitaxel compared to the nominal concentration, which met the requirements (Table 2).

**Table 2:** Stability of paclitaxel concentration in cell lysate and culture medium (*n* = 5)

Analyte nominal concentration (ng/mL)	Conditions	Measured concentration (Mean ± SD)	Accuracy (RE%)	Precision (RSD%)
1	4 h, ambient	1.07 ± 0.12	7.00	10.84
4000		3946.38 ± 176.19	1.34	4.45
1	3 freeze-thaw cycles (−20°C/ambient)	1.09 ± 0.12	9.20	11.30
4000		3959.60 ± 206.41	1.01	5.21
1	−80°C/30 days	1.06 ± 0.13	6.40	11.94
4000		4042.02 ± 212.51	1.05	1.05

Note: SD, standard deviation; RE, relative error; RSD, relative standard deviation.

### 3.3 Determination of Cellular Efflux of Paclitaxel from A549 and A549/TAX Cells at Different Paclitaxel Concentrations

**Figure 3:**
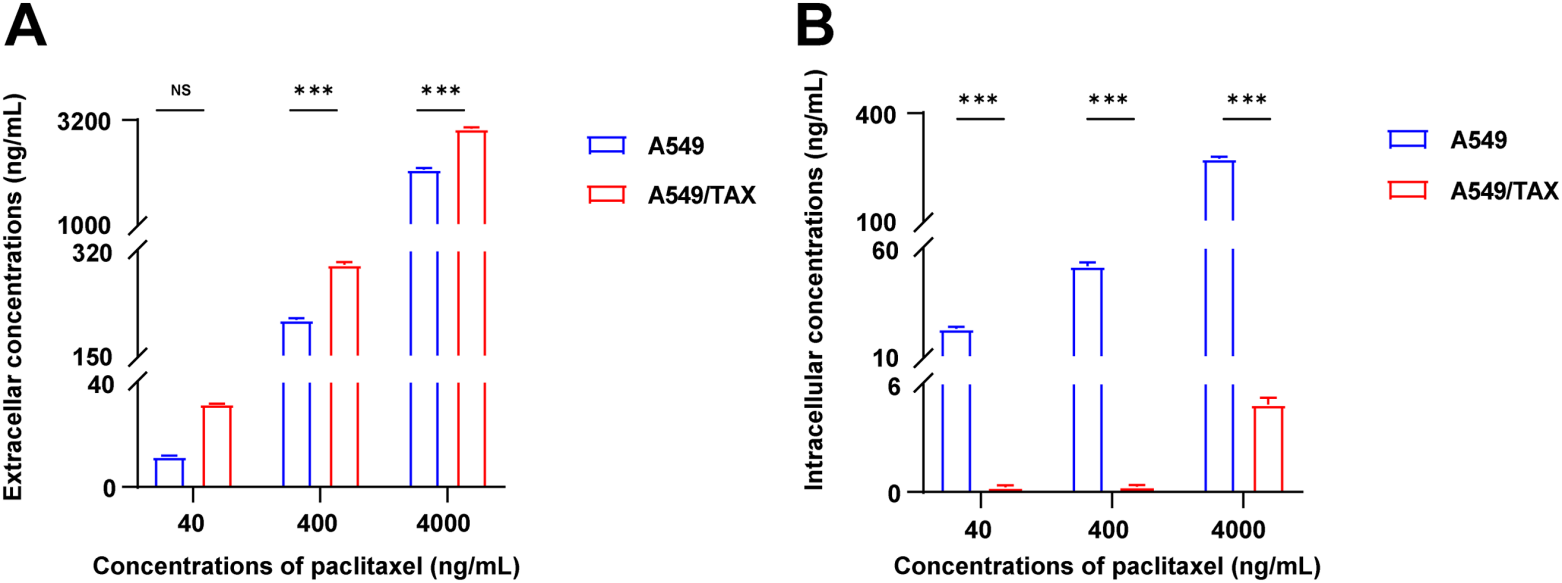
Determination of paclitaxel concentrations in A549 and A549/TAX cells and their culture media. (**A**) Paclitaxel concentration in the supernatants of the cell culture media of A549 and A549/TAX cells (*n* = 3). (**B**) Paclitaxel concentration in the cell pellets of A549 and A549/TAX cells (*n* = 3). ***p < 0.001. NS = not significant

Based on the aforementioned experiments, the findings indicate that ABCB1 is the principal mediator of paclitaxel resistance in NSCLC cells. To further elucidate the role of ABCB1-encoded P-glycoprotein (P-gp) in mediating paclitaxel transport, we indirectly assessed its activity by quantifying the intracellular and extracellular concentrations of paclitaxel. To clarify how ABCB1 mediates paclitaxel resistance, we established a UPLC-MS/MS method to quantify paclitaxel concentrations. At equivalent paclitaxel concentrations, paclitaxel levels in the supernatants of A549/TAX cells were higher than those in the supernatants of A549 cells, whereas the intracellular paclitaxel concentration in A549/TAX cells was significantly lower than that in A549 cells (Fig. 3A,B; Tables 3 and 4). These results indicate that the high expression of P-gp in A549/TAX cells may facilitate paclitaxel efflux from the intracellular to the extracellular compartment. Moreover, our data revealed that P-gp in A549/TAX cells maintained its transport function across a wide range of paclitaxel concentrations, from 80 to 8000 ng, suggesting that this transporter can mediate cellular efflux of at least 8000 ng of paclitaxel.

**Table 3:** UPLC-MS/MS analysis results for the supernatants of A549 and A549/TAX cells at different paclitaxel concentrations (*n* = 3, mean ± SD)

Cells	Paclitaxel Concentrations (ng/mL)		
	40	400	4000
A549	11.73 ± 0.35	208.30 ± 2.71	2156.30 ± 29.29
A549/TAX	31.91 ± 0.12	298.67 ± 3.86	3021.63 ± 24.80

**Table 4:** UPLC-MS/MS analysis results for the cell pellets of A549 and A549/TAX cells at different paclitaxel concentrations (*n* = 3, mean ± SD)

Cells	Paclitaxel Concentrations (ng/mL)		
	40	400	4000
A549	22.83 ± 0.85	52.03 ± 1.65	273.20 ± 5.05
A549/TAX	0.27 ± 0.12	0.30 ± 0.10	4.90 ± 0.36

### 3.4 Knockdown of ABCB1 Sensitizes Paclitaxel-Resistant NSCLC Cells

**Figure 4:**
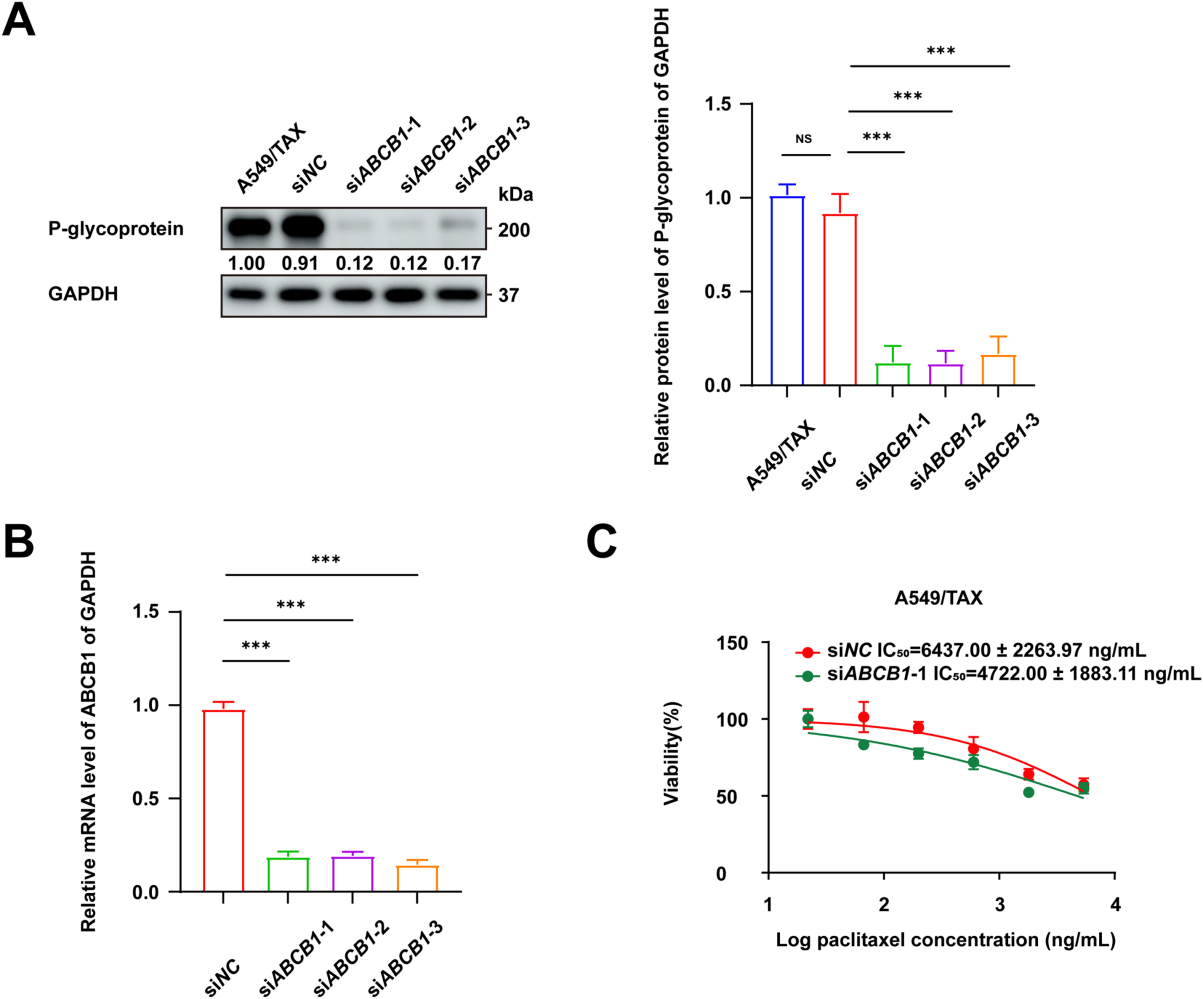

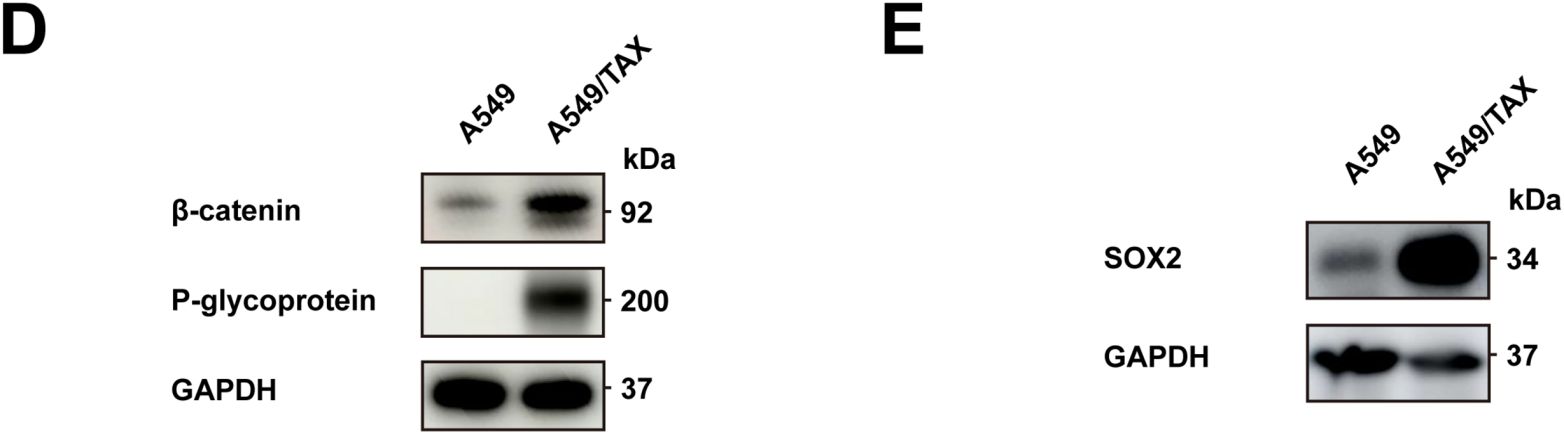
Knockdown of ABCB1 sensitizes paclitaxel-resistant NSCLC cells. (**A**) WB assay of P-gp expression in A549/TAX cells transfected with siNC or siABCB1 (*n* = 3). (**B**) qRT-PCR analysis of the mRNA levels of ABCB1 transfected with siNC or siABCB1 (*n* = 3). (**C**) CCK-8 assay results showing the efficacy of paclitaxel in A549/TAX cells transfected with siABCB1 or siNC for 72 h (*n* = 3). (**D**) WB assay of β-catenin and P-gp expression in NSCLC cells. (**E**) WB assay of SOX2 expression in NSCLC cells. ***p < 0.001. NS = not significant

Previous findings revealed a positive correlation between ABCB1 expression and paclitaxel resistance in NSCLC cells. The functional role of ABCB1 in mediating this resistance was further confirmed by knockdown assays in A549/TAX cells. The knockdown efficiency of three independent siRNAs targeting ABCB1 was validated by WB assay and qRT-PCR (Fig. 4A,B). Notably, transfection with siABCB1-1 reduced the IC_50_ value of A549/TAX cells from 6437.00 ± 2263.97 ng/mL to 4722.00 ± 1883.11 ng/mL (Fig. 4C). The results of CCK-8 assay showed that ABCB1 significantly contributes to paclitaxel resistance, and that its suppression effectively restores the sensitivity of NSCLC cells to paclitaxel. Given the pivotal role of ABCB1 in paclitaxel resistance of NSCLC cells, we further examined its upstream transcriptional regulators. We noted that β-catenin expression was markedly elevated in A549/TAX cells compared to that in A549 cells (Fig. 4D). Previous studies have also reported that the Wnt/β-catenin signaling pathway regulates ABCB1 transcription in cancer [27], suggesting that β-catenin may act as a critical upstream regulator of ABCB1. As cancer stemness is closely associated with chemoresistance, we assessed the expression of the stemness-related factor SOX2 in both cell lines. The results showed significantly higher SOX2 expression in A549/TAX cells (Fig. 4E), indicating that ABCB1 may contribute to paclitaxel resistance at least in part through a SOX2-mediated mechanism.

### 3.5 Synergistic Effect of P-gp Inhibitors and Paclitaxel

**Figure 5:**
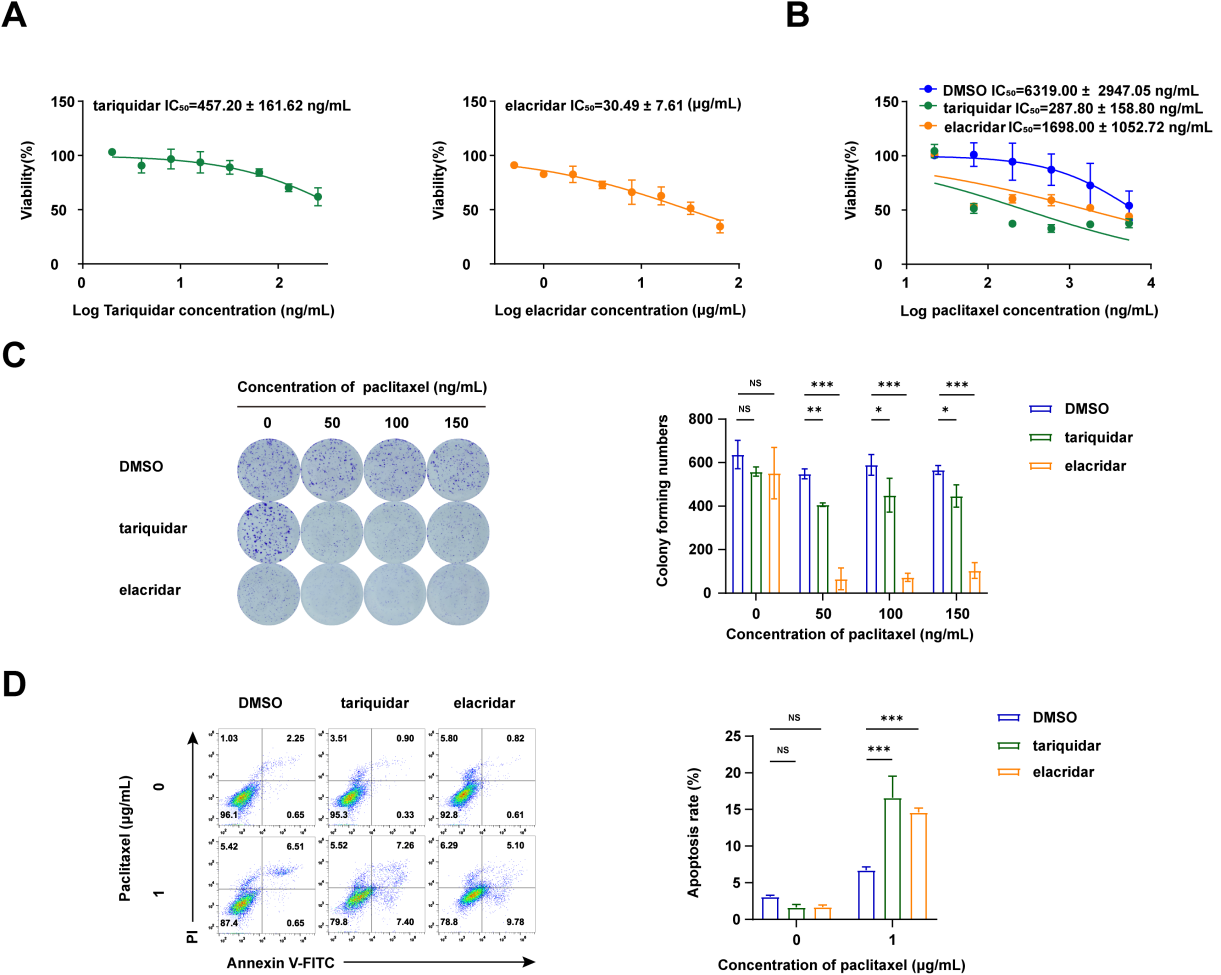
Synergistic effect of the P-gp inhibitors and paclitaxel. (**A**) Toxicity and safety of ABCB1 inhibitors (tariquidar and elacridar) were detected by CCK-8 assay (*n* = 3). (**B**) IC_50_ values of the combination of ABCB1 inhibitors (25 ng/mL tariquidar or 1.5 μg/mL elacridar) and paclitaxel were detected by CCK-8 assay (*n* = 3). (**C**) Colony formation assay of ABCB1 inhibitors (25 ng/mL tariquidar or 1.5 μg/mL elacridar) combined with paclitaxel (*n* = 3). (**D**) Apoptosis assay of ABCB1 inhibitors (25 ng/mL tariquidar or 1.5 μg/mL elacridar) combined with paclitaxel (*n* = 3). *p < 0.05, **p < 0.01, ***p < 0.001. NS = not significant

We then evaluated the combined effects of ABCB1 inhibitors (tariquidar and elacridar) with paclitaxel on A549/TAX cells. The toxicity of tariquidar and elacridar was assessed by CCK-8 assay (Fig. 5A), and nontoxic concentrations of tariquidar (25 ng/mL) and elacridar (1.5 μg/mL) were selected for subsequent experiments. The CCK-8 viability assay showed that elacridar reduced the IC_50_ value of paclitaxel on A549/TAX cells from 6319.00 ± 2947.05 ng/mL to 1698.00 ± 1052.72 ng/mL—a 3-fold reduction, while tariquidar similarly reduced the IC_50_ value to 287.80 ± 158.80 ng/mL—an approximately 21-fold reduction. Both these specific inhibitors lowered the IC_50_ value of paclitaxel on A549/TAX cells (Fig. 5B). Colony formation assays indicated that the paclitaxel-resistant cell group and the experimental groups treated with the two inhibitors showed no significant difference in the number of colonies in the absence of paclitaxel treatment. An increase in paclitaxel concentration from 50 to 150 ng/mL showed no effect on the proliferation capacity of resistant cells; however, the addition of tariquidar (25 ng/mL) and elacridar (1.5 μg/mL) significantly reduced cell proliferation (Fig. 5C). Apoptosis assays indicated that, the ABCB1 inhibitors enhanced paclitaxel-induced cytotoxicity in A549/TAX cells with paclitaxel at 1 μg/mL (Fig. 5D). Collectively, these results demonstrate that tariquidar and elacridar significantly reverse paclitaxel chemoresistance in A549/TAX cells by inhibiting P-gp function.

### 3.6 Determination of Cellular Efflux of Paclitaxel from A549/TAX Cells under P-gp Inhibition at Different Paclitaxel Concentrations

**Figure 6:**
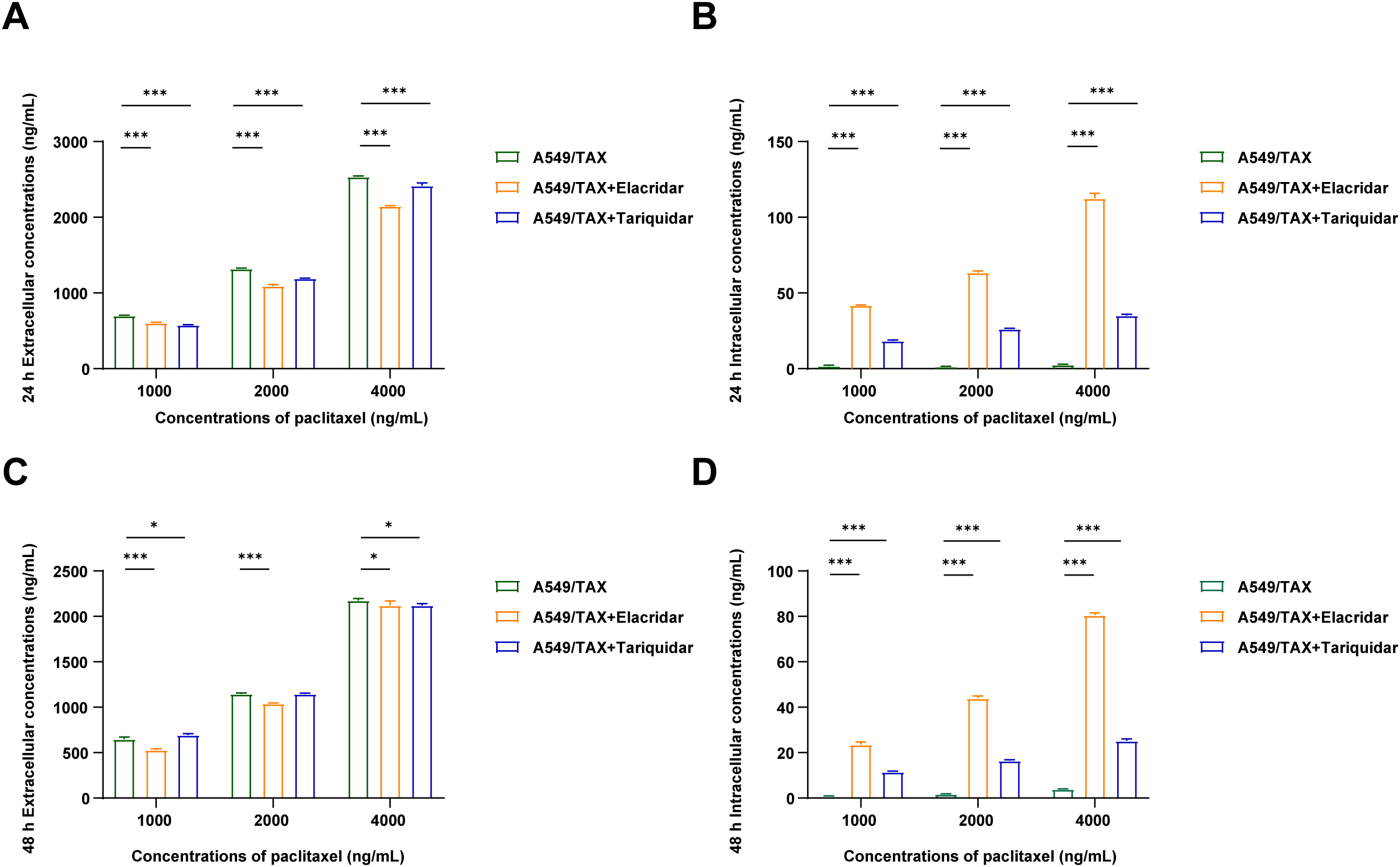
Effects of the combination of P-gp inhibitor with paclitaxel in A549 and A549/TAX cells and their culture media. (**A**) Paclitaxel concentration in cell culture supernatants was detected by UPLC-MS/MS following the treatment of A549/TAX cells with elacridar (1.5 μg/mL) or tariquidar (25 ng/mL) and different paclitaxel concentrations for 24 h (*n* = 3). (**B**) Paclitaxel concentration in cell pellets was detected by UPLC-MS/MS following the treatment of A549/TAX cells with elacridar (1.5 μg/mL) or tariquidar (25 ng/mL) and different paclitaxel concentrations for 24 h (*n* = 3). (**C**) Paclitaxel concentration in cell culture supernatants was detected by UPLC-MS/MS following the treatment of A549/TAX cells with elacridar (1.5 μg/mL) or tariquidar (25 ng/mL) and different paclitaxel concentrations for 48 h (*n* = 3). (**D**) Paclitaxel concentration in cell pellets was detected by UPLC-MS/MS following the treatment of A549/TAX cells with elacridar (1.5 μg/mL) or tariquidar (25 ng/mL) and different paclitaxel concentrations for 48 h (*n* = 3). *p < 0.05, ***p < 0.001

UPLC-MS/MS was conducted to quantify the changes in intracellular and extracellular paclitaxel concentrations under varying conditions of ABCB1 inhibitor treatment and paclitaxel doses. First, the cells were treated for 24 h with the combination of inhibitors and different paclitaxel concentrations; we observed that paclitaxel concentration decreased in the culture supernatants and increased in the cell pellets following tariquidar and elacridar addition. Moreover, 1.5 μg/mL elacridar was twice effective in augmenting intracellular paclitaxel concentration compared to 25 ng/mL tariquidar (Fig. 6A,B; Tables 5 and 6). Next, the cells were treated for 48 h with the combination of inhibitors and different paclitaxel concentrations. Interestingly, paclitaxel concentration in the cell culture supernatant slightly decreased over time in A549/TAX cells. Nevertheless, the addition of inhibitors decreased the concentration of paclitaxel in the cell culture supernatant. In contrast, the intracellular paclitaxel concentration in A549/TAX cells increased from 24 to 48 h, in accordance with the reduction in paclitaxel concentration in cell culture supernatant from 24 to 48 h as determined by UPLC-MS/MS (Fig. 6C,D; Tables 7 and 8). Paclitaxel concentration in cell pellets increased following P-gp inhibitor addition, although the extent of increase was lower than that in the 24 h group, suggesting that the therapeutic effect of the inhibitors diminished over time. Taken together, these results suggest that elacridar and tariquidar play a role in decreasing the efflux of paclitaxel while increasing the intracellular accumulation of paclitaxel; moreover, the effectiveness of 1.5 μg/mL elacridar in inhibiting P-gp was considerably higher than that of 25 ng/mL tariquidar. To further validate these findings, we established ABCB1 knockdown cell lines and examined the intracellular and extracellular concentrations of paclitaxel following drug treatment. Consistent with the results obtained from inhibitor intervention, ABCB1 silencing reduced extracellular paclitaxel concentrations while enhancing intracellular drug accumulation (Tables A1 and A2). These results provide additional evidence that ABCB1 plays a critical role in mediating paclitaxel transport and resistance in A549/TAX cells.

**Table 5:** UPLC-MS/MS-based detection of paclitaxel concentration in the cell culture supernatant following the treatment of A549/TAX cells with elacridar (1.5 μg/mL) or tariquidar (25 ng/mL) and different paclitaxel concentrations for 24 h (*n* = 3, mean ± SD)

Cells	Paclitaxel Concentration (ng/mL)		
	1000	2000	4000
A549/TAX	701.00 ± 6.36	1320.63 ± 7.54	2536.33 ± 9.56
A549/TAX + elacridar	606.43 ± 6.79	1094.43 ± 17.04	2149.67 ± 3.75
A549/TAX + tariquidar	578.57 ± 2.14	1194.03 ± 2.50	2418.57 ± 34.06

**Table 6:** UPLC-MS/MS-based detection of paclitaxel concentration in the cell pellets following the treatment of A549/TAX cells with elacridar (1.5 μg/mL) or tariquidar (25 ng/mL) and different paclitaxel concentrations for 24 h (*n* = 3, mean ± SD)

Cells	Paclitaxel Concentration (ng/mL)		
	1000	2000	4000
A549/TAX	1.83 ± 0.49	1.47 ± 0.06	2.67 ± 0.31
A549/TAX + elacridar	41.90 ± 0.20	63.53 ± 1.02	112.53 ± 3.29
A549/TAX + tariquidar	18.47 ± 0.51	26.30 ± 0.36	35.23 ± 0.70

**Table 7:** UPLC-MS/MS-based detection of paclitaxel concentration in the cell culture supernatants following the treatment of A549/TAX cells with elacridar (1.5 μg/mL) or tariquidar (25 ng/mL) and different paclitaxel concentrations for 48 h (*n* = 3, mean ± SD)

Cells	Paclitaxel Concentration (ng/mL)		
	1000	2000	4000
A549/TAX	649.17 ± 22.04	1147.57 ± 9.24	2176.93 ± 18.93
A549/TAX + elacridar	529.47 ± 12.33	1040.30 ± 6.87	2122.43 ± 47.09
A549/TAX + tariquidar	693.87 ± 15.50	1147.60 ± 8.39	2121.80 ± 19.75

**Table 8:** UPLC-MS/MS-based detection of paclitaxel concentration in the cell pellets following the treatment of A549/TAX cells with elacridar (1.5 μg/mL) or tariquidar (25 ng/mL) and different paclitaxel concentrations for 48 h (*n* = 3, mean ± SD)

Cells	Paclitaxel Concentration (ng/mL)		
	1000	2000	4000
A549/TAX	0.83 ± 0.15	1.77 ± 0.12	3.93 ± 0.12
A549/TAX + elacridar	23.57 ± 1.10	43.97 ± 0.95	80.50 ± 1.00
A549/TAX + tariquidar	11.53 ± 0.32	16.43 ± 0.45	25.20 ± 0.87

## 4 Discussion

Paclitaxel is one of the most clinically effective anticancer agents and is the primary first-line therapy for advanced NSCLC, particularly in patients lacking actionable genetic mutations [28]. However, the prolonged administration of paclitaxel frequently results in acquired resistance, substantially limiting its therapeutic benefit. Therefore, it is crucial to elucidate the molecular mechanisms underlying paclitaxel resistance and develop strategies to overcome it. Among the potential targets, ABCB1 has emerged as a promising candidate for reversing paclitaxel resistance.

In the present study, we observed that paclitaxel-resistant cells exhibited markedly elevated expression of ABCB1. By using UPLC-MS/MS, we further quantified intracellular and extracellular paclitaxel concentrations in parental (A549) and resistant (A549/TAX) cells; the results confirmed that resistance to paclitaxel is primarily attributable to the drug efflux function of P-gp. Notably, ABCB1 knockdown significantly enhanced the cytotoxicity of paclitaxel, thereby establishing the pivotal role of P-gp in mediating paclitaxel resistance and highlighting the therapeutic potential of its inhibition. Furthermore, our study also utilized human NSCLC specimens rather than animal models, thereby increasing translational relevance. We demonstrated that ABCB1 overexpression in clinical samples is closely associated with paclitaxel resistance and adverse prognosis.

Consistent with these findings, the administration of P-gp inhibitors substantially augmented paclitaxel-induced cytotoxicity, indicating a promising strategy for NSCLC treatment. P-gp is an ATP-dependent efflux transporter with broad substrate specificity, and numerous studies have demonstrated that its inhibition enhances chemosensitivity across multiple malignancies. For example, Creemers et al. [29] reported that tariquidar sensitized adrenocortical carcinoma cells to doxorubicin and etoposide, while Lagas et al. [30] showed that elacridar increased dasatinib accumulation in the brain. Similarly, Joshi et al. [31] identified natural alkaloid compounds that can effectively inhibit P-gp activity.

Despite encouraging preclinical results, the clinical translation of ABCB1 inhibitors faces substantial challenges. First-generation inhibitors such as verapamil and cyclosporine A partially reversed multidrug resistance (MDR) in early clinical trials; however, their clinical application was impeded by substantial toxicity at effective doses, including severe hypotension and immunosuppression. Second-generation inhibitors, including valspodar and biricodar, were designed with improved specificity and reduced toxicity; however, pivotal Phase III trials—such as those combining valspodar with paclitaxel for treating ovarian cancer—failed to demonstrate survival benefits, largely because of complex pharmacokinetic interactions. Third-generation inhibitors (e.g., tariquidar, zosuquidar, and laniquidar) demonstrated superior selectivity and potency with reduced off-target effects; nevertheless, large-scale Phase III trials in patients with ovarian cancer and NSCLC again yielded disappointing outcomes, failing to improve overall survival [32,33]. These limitations highlight the multifactorial nature of MDR, the challenge of achieving effective intratumoral inhibitor concentrations without systemic toxicity, and the necessity of better patient stratification. Notably, the high expression of P-gp at the blood–brain barrier has received increasing attention of researchers [34]. The inhibition of P-gp may facilitate enhanced penetration of diverse agents into the central nervous system, including chemotherapeutics for glioblastoma and drugs for Alzheimer’s disease and epilepsy. For example, recent studies have explored the combination of tariquidar with temozolomide to improve drug delivery in patients with glioblastoma [35,36]. Although promising, such strategies require rigorous clinical evaluation to establish both efficacy and safety. Overall, despite decades of clinical research, no P-gp inhibitor has yet been approved for clinical MDR reversal because of these unresolved challenges.

This study has several limitations. First, the synergistic effect of combining ABCB1 inhibitors with paclitaxel was demonstrated only in vitro. Further in vivo experiments using animal models are necessary to verify the therapeutic efficacy and safety of this combination strategy. In particular, UPLC-MS/MS could be applied to quantitatively monitor paclitaxel distribution and pharmacokinetic changes in vivo, providing stronger evidence for the inhibitory mechanism of ABCB1. Second, the number of clinical samples analyzed in this study was relatively limited. Expanding the sample size and including patients with different clinicopathological characteristics would help to validate the clinical relevance of ABCB1 overexpression and to assess its potential as a predictive biomarker for paclitaxel resistance in NSCLC.

**Figure 7:**
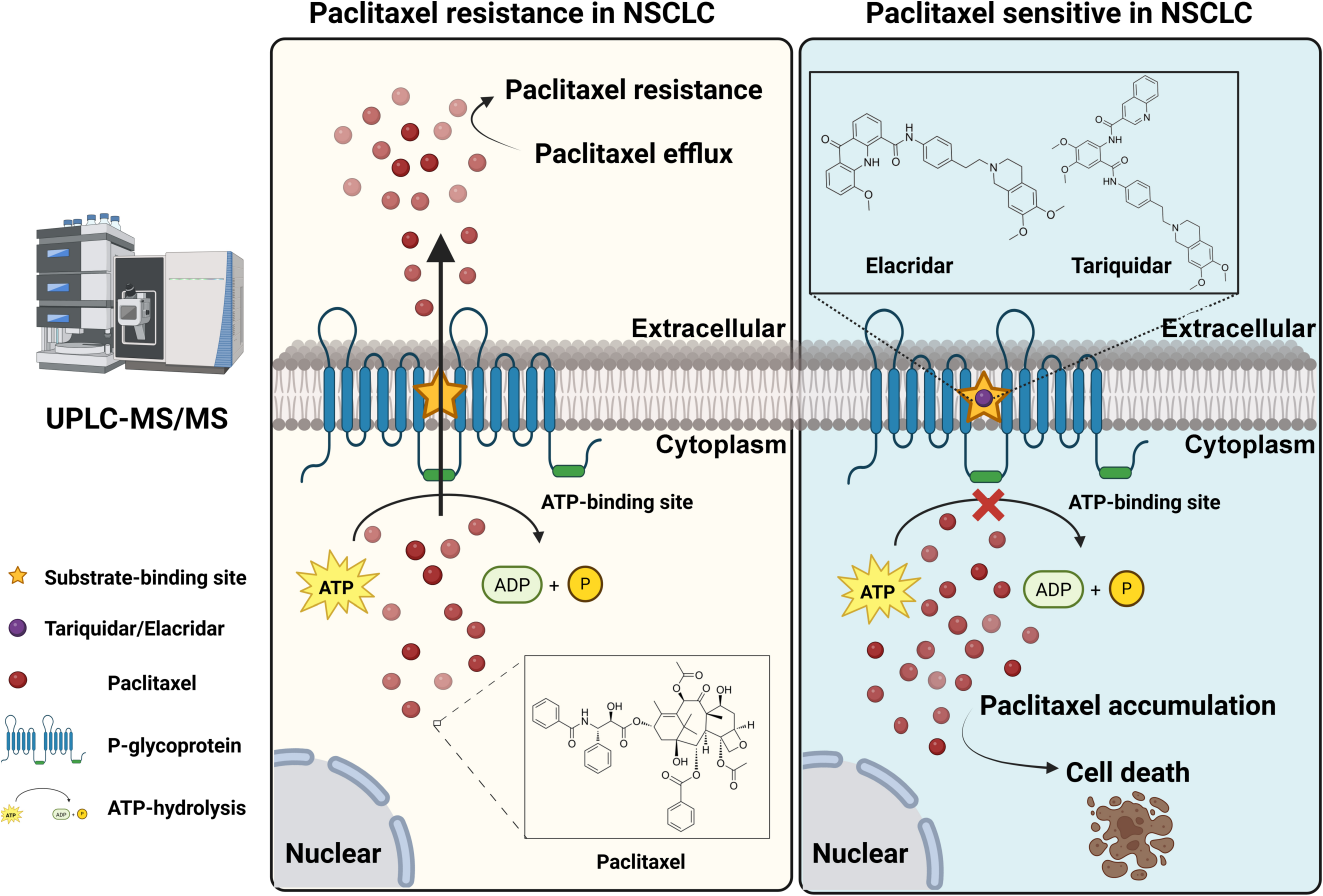
A model depicting ABCB1-regulated paclitaxel resistance in NSCLC cells and detection by the UPLC/MS-MS method

In summary, although the clinical application of P-gp inhibitors is currently limited, our findings reinforce the central role of ABCB1 in NSCLC chemoresistance and highlight its potential as a therapeutic target. We further demonstrated the utility of UPLC-MS/MS for quantifying intracellular paclitaxel concentrations, offering a valuable methodological advancement for resistance mechanism studies (Fig. 7). Collectively, these results provide a rationale for continued efforts to optimize P-gp-targeted therapeutic strategies and for future clinical trials aimed at overcoming ABCB1-mediated paclitaxel resistance in NSCLC cells.

## Data Availability

The data that support the findings of this study are available from the corresponding authors, upon reasonable request.

## Appendix A

**Table A1:** The supernatant of A549/TAX cells of siNC and siABCB1-1 at different concentrations was analyzed by UPLC-MS/MS for 48 h

Cells	Concentrations of paclitaxel (ng/mL)	
	40	400
siNC	7.70	49.00
siABCB1-1	5.10	45.20

**Table A2:** The cell pellets of A549/TAX cells of siNC and siABCB1-1 at different concentrations by UPLC-MS/MS for 48 h

Cells	Concentrations of paclitaxel (ng/mL)	
	40	400
siNC	0.30	0.20
siABCB1-1	0.50	1.00
